# Serotypes and antimicrobial resistance profiles of *Salmonella enterica* recovered from clinical swine samples

**DOI:** 10.14202/vetworld.2020.2312-2318

**Published:** 2020-11-03

**Authors:** Siriporn Kongsoi, Suksun Chumsing, Darunee Satorn, Panisa Noourai

**Affiliations:** 1 Department of Veterinary Public Health, Faculty of Veterinary Medicine, Kasetsart University, Nakhon Pathom 73140, Thailand; 2 Department of Microbiology, Faculty of Public Health, Mahidol University, Bangkok 10400, Thailand

**Keywords:** clinical isolates, multidrug resistance, *Salmonella* Choleraesuis

## Abstract

**Background and Aim:**

*Salmonella enterica* is an important foodborne pathogen and is recognized as a major public health issue. The emergence of multidrug-resistant (MDR) *S. enterica* represents a major challenge for national public health authorities. We investigated the distribution of serovars and antimicrobial resistance of *S. enterica* isolates from clinical swine samples stored at the Veterinary Diagnostic Laboratory, Faculty of Veterinary Medicine, Kasetsart University from 2016 to 2017.

**Materials and Methods:**

Clinical samples were collected and subjected to standard microbiological techniques outlined in the Manual of Clinical Microbiology to identify *Salmonella* serovars. Susceptibility to antimicrobials was tested by the Kirby–Bauer disk diffusion method using a panel of 14 antimicrobials.

**Results:**

A total of 144 *Salmonella* isolates were identified and the dominant serovar was *Salmonella* Choleraesuis (66.67%), followed by monophasic *Salmonella* Typhimurium (18.75%), S. Typhimurium (9.03%), and Rissen (5.56%). The isolates displayed high resistance rates to ampicillin (AMP [100%]), amoxicillin (AX [100%]), tetracycline (TE [100%]), cefotaxime (CTX [89.58%]), ceftriaxone (CRO [87.50%]), chloramphenicol (C [82.64%]), gentamicin (CN [79.17%]), nalidixic acid (NA [72.92%]), and ceftazidime (CAZ [71.53%]). All isolates were MDR, with 29 distinct resistance patterns. The dominant MDR pattern among serovars Choleraesuis and Rissen exhibited resistance to 9 antimicrobials: (R7-14 AMP-AX-CAZ-CRO-CTX-NA-C-CN-TE). However, all tested isolates were susceptible to AX/clavulanic acid and fosfomycin.

**Conclusion:**

High resistance levels to the third generation of cephalosporins such as CAZ, CRO, and CTX highlight the need for careful and reasonable usage of antimicrobials in animals and humans, especially for *S*. Choleraesuis infections.

## Introduction

*Salmonella enterica* is a major foodborne zoonotic agent associated with industrially produced food animals and is thus a public health concern in many countries [1]. *S. enterica* is one of the important enteric pathogens in swine, causing enterocolitis and/or acute septicemia in animals of all ages, particularly in weaned and nursery swine [2-4]. Symptoms of salmonellosis in swine vary widely from reduced weight gain to sudden death, all of which translate to economic losses in the swine industry worldwide [2-5].

Swine are considered an important reservoir host for many serovars of *Salmonella* and contaminated pork is a known source of human infections [6,7]. Antimicrobials have been used in swine for therapeutic purposes to treat disease, for prophylactic or metaphylactic purposes to prevent disease, and for growth promotion [8]. Due to the overuse of antimicrobials in recent years, the rate of resistant and even multidrug-resistant (MDR) *Salmonella* has risen dramatically, resulting in more frequent failures in the treatment of human salmonellosis [9-12]. The potential for treatment failures, especially in life-threatening situations, as a result of antimicrobial-resistant *Salmonella* infections highlights the need for further research and surveillance targeting *Salmonella* in the pork production chain. In particular, information on the distribution of antimicrobial resistance in *S. enterica* serovars among healthy and clinically affected swine is important to public health. Several studies have investigated the incidence of antimicrobial-resistant *Salmonella* carriage in apparently healthy swine from finishing herds [13-15]. However, there has been relatively limited research on the serotype diversity of resistant *S. enterica* isolates recovered from clinically diseased swine. As mentioned above, there is a need for more information on the distribution of antimicrobial resistance in *S. enterica* serovars among healthy and clinically affected swine. Clinical isolates are especially important for antimicrobial resistance monitoring and surveillance efforts because clinically ill animals receive more antimicrobial treatments; therefore, these clinical isolates have potentially encountered the most antimicrobial selective pressure.

The objectives of this study were to examine the serovar distribution and antimicrobial resistance among *Salmonella* isolates recovered from various clinical samples of swine submitted to the Veterinary Diagnostic Laboratory, Faculty of Veterinary Medicine, Kasetsart University, Thailand. Our approach can be the basis for developing an early warning system for emerging pathogens and antimicrobial resistance threats. Our results will provide important information to develop management strategies and policy decisions on the monitoring and surveillance of *Salmonella* in food animals.

## Materials and Methods

### Ethical approval

Ethical approval was not necessary for this study.

### Study location, period and sample collection

The Veterinary Diagnostic Laboratory, Faculty of Veterinary Medicine, Kasetsart University receives clinical samples (whole animals, carcasses, swabs, tissues, and organs) predominantly from swine farms within Nakhon Pathom and Ratchaburi, the two largest pig-producing provinces in Thailand. All clinical samples originally collected by postmortem examination (necropsy) and delivered to the laboratory for diagnostic purposes, and thus no experimental investigation on swine was performed. All swine clinical samples from July 2016 and through August 2017 were included in this study.

### Isolation and Identification

Clinical samples consisted of materials submitted for isolating and identifying *Salmonella* in the course of routine diagnostic procedures. The clinical samples were directly plated onto MacConkey agar (Oxoid, UK) and xylose lysine deoxycholate (Oxoid, UK) agar and incubated for 24 h at 37°C. The plates were examined for typical *Salmonella* colonies and up to five presumptive *Salmonella* colonies were selected and identified biochemically. Various biochemical media were inoculated and incubated overnight at 37°C, and the results were observed and interpreted [16].

### Serotyping

The serogroup of each isolate was identified using BD Difco™ *Salmonella* O antisera (Becton Dickinson, US) at the Veterinary Diagnostic Laboratory, Nakhon Pathom. The serotype was determined according to the White-Kauffmann-Le Minor scheme [17,18], which involves slide agglutination to define O and H antigens using commercial antisera obtained from the S&A Reagents Lab, Bangkok.

### Antimicrobial susceptibility testing

The antimicrobial susceptibilities of *Salmonella* isolates to 14 types of antimicrobials were determined by the standard Kirby–Bauer disk diffusion method according to the guidelines and interpretative criteria of the Clinical and Laboratory Standards Institute (CLSI, 2017) [19]. The following antimicrobial disks (Oxoid, UK) were used in this study: Ampicillin (AMP, 10 μg), amoxicillin (AX, 25 μg), AX/clavulanic acid (AMC, 20/10 μg), cefotaxime (CTX, 30 μg), ceftriaxone (CRO, 30 μg), ceftazidime (CAZ, 30 μg), gentamicin (CN, 10 μg), tetracycline (TE, 30 μg), chloramphenicol (C, 30 μg), nalidixic acid (NA, 30 μg), ciprofloxacin (CIP, 5 μg), trimethoprim/sulfamethoxazole (SXT, 1.25/23.75 μg), colistin (CT, 10 μg), and fosfomycin (FOT, 200 μg). The reference strain, *Escherichia coli* ATCC 25922, was used as a control. According to the CLSI, each isolate was evaluated as either susceptible, intermediate, or resistant. In addition, *Salmonella* isolate that is resistant to at least three antimicrobial classes was defined as an MDR *Salmonella* isolate.

## Results

### Prevalence and serovar distribution

We processed a total of 337 clinical samples from 163 pigs in 46 farms, yielding a total of 144 *Salmonella* isolates that were recovered and identified from 52 (15.43%) clinical samples. The isolation rates of *Salmonella* were 77.78% (7/9) in spleen samples, 50.00% (14/28) in mesenteric lymph node (MSLN) samples, 14.29% (20/140) in lung samples, 13.33% (6/45) in intestine samples, 8.33% (1/12) in brain swab samples, 4.65% (2/43) in joint swab samples, 3.45% (1/29) in tracheobronchial lymph node (TBLN) samples, and 3.23% (1/31) in tonsil samples.

**Figure-1:**
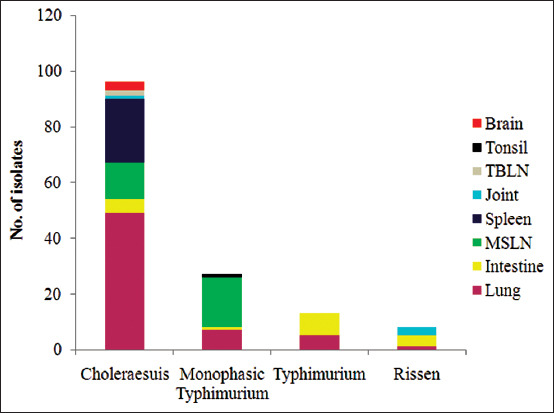
The distribution of *Salmonella* serovars in various clinical samples.

We identified four serovars: *Salmonella* Choleraesuis (96/144, 66.67%) was the most predominant, followed by monophasic *Salmonella* Typhimurium (27/144, 18.75%), *S*. Typhimurium (13/144, 9.03%), and *S*. Rissen (8/144, 5.56%). The distribution of these serovars among the different clinical samples is given in Figure-1. Serovar Choleraesuis was most prevalent in lung samples (49/96, 51.04%), followed by spleen samples (23/96, 23.96%), MSLN samples (13/96, 13.54%), intestine samples (5/96, 5.21%), brain swab samples (3/96, 3.13%), TBLN samples (2/96, 2.08%), and joint samples (1/96, 1.04%).

Serovar monophasic Typhimurium was isolated from MSLN (18/27, 66.67%), lung (7/27, 25.93%), intestine (1/27, 3.70%), and tonsil (1/27, 3.70%) samples. Serovar Typhimurium was detected in intestine (8/13, 61.54%) and lung (5/13, 38.46%) samples, while serovar Rissen was isolated from intestine (4/8, 50.00%), joint swab (3/8, 37.50%), and lung (1/8, 12.50%) samples.

### Antimicrobial resistance and multidrug resistance patterns

Antimicrobial susceptibility testing of the 144 isolates showed that they were all sensitive to AMC acid and FOT; and they were all resistant to AMP, AX, and TX. The following antibiotics also elicited resistance among the isolates (arranged in decreasing frequency of resistant isolates): Cefotaxime (129, 89.58%), followed by ceftriaxone (126 isolates, 87.50%), chloramphenicol (119, 82.64%), gentamicin (114, 79.17%), nalidixic acid (105, 72.92%), ceftazidime (103, 71.53%), trimethoprim/sulfamethoxazole (21, 14.58%), colistin (20, 13.89%), and ciprofloxacin (15, 10.42%). In addition, the following antibiotics elicited intermediate resistance among the isolates: Ciprofloxacin (112, 77.78%), trimethoprim/sulfamethoxazole (52, 36.11%), ceftazidime (19, 13.19%), nalidixic acid (7, 4.68%), and ceftriaxone (3, 2.08%). Furthermore, the isolates were most susceptible to colistin (124, 86.11%), followed by trimethoprim/sulfamethoxazole (71, 49.31%), nalidixic acid (32, 22.22%), gentamicin (30, 20.83%), chloramphenicol (25, 17.36%), ceftazidime (22, 15.28%), ciprofloxacin (17, 11.81%), ceftriaxone (15, 10.42%), and cefotaxime (15, 10.42%).

**Figure-2:**
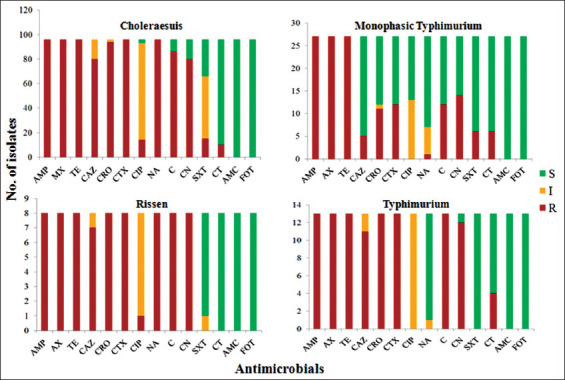
Antimicrobial resistance diversity of each *Salmonella* serovar. Red indicates resistance (R) to the corresponding antimicrobials, yellow indicates intermediate (I) resistance, and green indicates susceptible (S) phenotype. The names of the antimicrobials are abbreviated as ampicillin (AMP), amoxicillin (AX), tetracycline (TE), ceftazidime (CAZ), ceftriaxone (CRO), cefotaxime (CTX), ciprofloxacin (CIP), nalidixic acid (NA), chloramphenicol (C), gentamicin (CN), trimethoprim/sulfamethoxazole (SXT), colistin (CT), amoxicillin/clavulanic acid (AMC), and fosfomycin (FOT).

The prevalence levels of antibiotic resistance among *Salmonella* serovars are shown in Figure-2. For Choleraesuis isolates, 100% (96/96) were resistant to AMP, AX, TE, CTX, and NA; 97.92% (94/96) were resistant to CRO; 89.58% (86/96) were resistant to C; 83.33% (80/96) were resistant to CAZ and CN; 15.63% (15/96) were resistant to SXT; 14.58% (14/96) were resistant to CIP; and 10.42% (10/96) were resistant to CT.

For the monophasic serovar, 100% (27/27) were resistant to AMP, AX, and TE; 51.85% (14/27) were resistant to CN; 44.44% (12/27) were resistant to CTX and C; 40.74% (11/27) were resistant to CRO; 22.22% (6/27) were resistant to SXT and CT; 18.52% (5/27) were resistant to CAZ; and 3.70% (1/27) were resistant to NA.

For the Typhimurium isolates, 100% (13/13) were resistant to AMP, AX, TE, CRO, CTX, and C; 92.31% (12/13) were resistant to CN; 84.62% (11/13) were resistant to CAZ; and 30.77% (4/13) were resistant to CT.

For the Rissen isolates, 100% (8/8) were resistant to AMP, AX, TE, CRO, CTX, NA, C, and CN; 87.50% (7/8) were resistant to CAZ; and 12.5% (1/8) were resistant to CIP.

All of the 144 isolates were resistant to at least one antimicrobial and exhibited the MDR phenotype. The MDR patterns of the isolates listed in Table-1 show a wide spectrum of antimicrobial resistance. We found a total of 29 MDR patterns reflecting an individual isolate’s resistance to 3-9 antimicrobial classes. The MDR patterns with resistance to 9 classes of antimicrobials were observed only in *S*. Choleraesuis isolates. The most common MDR patterns among the serovars Choleraesuis and Rissen were R7-14 AMP-AX-CAZ-CRO-CTX-NA-C-CN-TE (36, 25.00%) followed by R7-18 AMP-AX-CRO-CTX-NA-C-CN-TE (15, 10.42%).

**Table-1:** The MDR patterns of *Salmonella* serovars.

Serovars	MDR patterns	No. of isolates (%)
Choleraesuis (n=96)	6 classes	21 (14.58)
	R6-23 AMP-AX-CAZ-CRO-CTX-NA-C-TE	13 (9.03)
	R6-22 AMP-AX-CAZ-CRO-CTX-NA-CN-TE	8 (5.56)
	7 classes	49 (34.03)
	R7-14 AMP-AX-CAZ-CRO-CTX-NA-C-CN-TE	30 (20.83)
	R7-18 AMP-AX-CRO-CTX-NA-C-CN-TE	14 (9.72)
	R7-21 AMP-AX-CAZ-CRO-CTX-CIP-NA-CN-TE	2 (1.39)
	R7-24 AMP-AX-CAZ-CTX-NA-CT-C-TE	2 (1.39)
	R7-26 AMP-AX-CAZ-CRO-CTX-NA-CT-C-TE	1 (0.69)
	8 classes	18 (12.50)
	R8-6 AMP-AX-CAZ-CRO-CTX-NA-C-CN-TE-SXT	8 (5.56)
	R8-15 AMP-AX-CAZ-CRO-CTX-NA-CT-C-CN-TE	5 (3.47)
	R8-16 AMP-AX-CAZ-CRO-CTX-CIP-NA-C-CN-TE	4 (2.78)
	R8-20 AMP-AX-CRO-CTX-NA-CT-C-CN-TE	1 (0.69)
	9 classes	8 (5.56)
	R9-19 AMP-AX-CAZ-CRO-CTX-CIP-NA-C-CN-TE-SXT	6 (4.17)
	R9-17 AMP-AX-CAZ-CRO-CTX-CIP-NA-CT-C-CN-TE	1 (0.69)
	R9-25 AMP-AX-CRO-CTX-CIP-NA-C-CN-TE-SXT	1 (0.69)
Monophasic Typhimurium (n=27)	3 classes	10 (6.94)
	R3-3 AMP-AX-TE	10 (6.94)
	5 classes	3 (2.08)
	R5-2 AMP-AX-CT-C-TE	1 (0.69)
	R5-4 AMP-AX-CRO-CTX-CN-TE	1 (0.69)
	R5-8 AMP-AX-C-TE-SXT	1 (0.69)
	6 classes	11 (7.64)
	R6-1 AMP-AX-CAZ-CRO-CTX-C-CN-TE	3 (2.08)
	R6-5 AMP-AX-CRO-CTX-CT-CN-TE	3 (2.08)
	R6-10 AMP-AX-CRO-CTX-C-CN-TE	2 (1.39)
	R6-7 AMP-AX-CT-C-TE-SXT	1 (0.69)
	R6-9 AMP-AX-CTX-C-CN-TE	1 (0.69)
	R6-12 AMP-AX-C-CN-TE-SXT	1 (0.69)
	7 classes	2 (1.39)
	R7-11 AMP-AX-CAZ-CRO-CTX-C-CN-TE-SXT	1 (0.69)
	R7-13 AMP-AX-CT-C-CN-TE-SXT	1 (0.69)
	8 classes	1 (0.69)
	R8-6 AMP-AX-CAZ-CRO-CTX-NA-C-CN-TE-SXT	1 (0.69)
Typhimurium (n=13)	6 classes	10 (6.94)
	R6-1 AMP-AX-CAZ-CRO-CTX-C-CN-TE	9 (6.25)
	R6-27 AMP-AX-CRO-CTX-CT-C-TE	1 (0.69)
	7 classes	3 (2.08)
	R7-29 AMP-AX-CAZ-CRO-CTX-CT-C-CN-TE	2 (1.39)
	R7-28 AMP-AX-CRO-CTX-CT-C-CN-TE	1 (0.69)
Rissen (n=8)	7 classes	7 (4.86)
	R7-14 AMP-AX-CAZ-CRO-CTX-NA-C-CN-TE	6 (4.17)
	R7-18 AMP-AX-CRO-CTX-NA-C-CN-TE	1 (0.69)
	8 classes	1 (0.69)
	R8-16 AMP-AX-CAZ-CRO-CTX-CIP-NA-C-CN-TE	1 (0.69)

MDR=Multidrug resistant, AMP=Ampicillin, AX=Amoxicillin, TE=Tetracycline, CTX=Cefotaxime, CRO=Ceftriaxone, C=Chloramphenicol, CN=Gentamicin, NA=Nalidixic acid, CAZ=Ceftazidime, CIP=Ciprofloxacin, SXT=Trimethoprim/sulfamethoxazole, CT=Colistin

## Discussion

This is the first report linking serovars and antimicrobial resistance in *Salmonella* isolates obtained from clinical swine samples in Thailand. Our results identify the serovars originating from food-producing animals that require greater attention.

Overall, the serovar diversity among the clinical isolates was low, that is, only four *Salmonella* serovars were detected: Choleraesuis, monophasic Typhimurium, Typhimurium, and Rissen. These serovars were associated with various clinical samples or systemic infections of diseased swine. *S*. Choleraesuis is highly adapted to swine and usually causes swine paratyphoid with clinical manifestations of enterocolitis and septicemia. In humans, it frequently causes septicemic disease with little or no involvement of the gastrointestinal tract [4]. In Thailand, *S*. Choleraesuis swine infections are never reported in any official database because they cause mild symptoms that have never been associated with an outbreak. Therefore, it has never been microbiologically investigated. Thus, it is difficult to obtain a true picture of the occurrence of *S*. Choleraesuis infections in swine populations in Thailand.

At present, *Salmonella* 1,4,[5],12:i:- is considered a monophasic variant of *S*. Typhimurium that lacks the second-phase flagellar antigen. It has emerged as a major public health concern in multiple countries worldwide, including Thailand [20-24]. Pigs and pork products have often been implicated in outbreaks of monophasic *S*. Typhimurium. In this study, monophasic Typhimurium was more prevalent than *S*. Typhimurium and *S*. Rissen. Our results are not consistent with previous findings in Thailand suggesting that *S*. Rissen and *S*. Typhimurium are the most common serovars in swine [25-27]. The lack of agreement between studies may be due to the differences in study design and sample types.

AMP, C, and SXT are traditional first-line antimicrobial agents that have been used for treating *Salmonella* infections in humans since before the 1980s. By the late 1980s, MDR *Salmonella* had been observed in many countries [28-31]. Recently, extended-spectrum cephalosporins and fluoroquinolones have been suggested as alternative treatment options for MDR *Salmonella* infections in humans [32,33]. This study demonstrated that all clinical isolates belonging to serovars Choleraesuis, monophasic Typhimurium, Typhimurium, and Rissen were MDR, with resistances to 3-9 antimicrobial classes and exhibiting 29 distinct MDR patterns. The extremely high frequency of MDR among *Salmonella* serovars isolated from clinical swine samples is a remarkable observation that should raise concern. In Thailand, only one study has reported on antimicrobial use in swine farming [34], this study reported on the following antimicrobials: Penicillin, AX, cephalexin, TE, sulfadimethoxine, sulfamerazine, enrofloxacin, CIP, lincomycin, CN, kanamycin, erythromycin, tiamulin, tylosin, and CT [34]. Their results are consistent with our findings regarding the most common MDR patterns. Collectively, these findings show that various MDR patterns in *Salmonella* serovars occur regardless of the level of overuse and misuse of antimicrobials during all stages of swine production.

This study also reports for the first time the high rate of resistance to third-generation cephalosporins (CAZ, CRO, and CTX) and reduced susceptibility to a fluoroquinolone (CIP) in *S*. Choleraesuis, *S*. Typhimurium, and *S*. Rissen obtained from clinical swine samples in Thailand. *S*. Choleraesuis, the most prevalent serovar in this study, is the second most common serovar implicated in human septicemia in Thailand [25,35,36]. In addition, recent studies have reported extremely high frequencies of CRO and fluoroquinolone resistance among invasive *S*. Choleraesuis isolated from bacteremic patients in Thailand [25,35,36]. Therefore, the chain of transmission and mechanism of resistance in MDR *S*. Choleraesuis should be further investigated to reduce the spread of resistance and its threat to human health in Thailand. It should be noted that although some *Salmonella* isolates were resistant to cephalosporins and fluoroquinolone in the present study, all isolates were susceptible to AMC acid and FOT.

This study was conducted as a passive surveillance on clinical samples that had been previously submitted for diagnostic purposes. Therefore, the results may not be representative of the general swine population in Thailand and potential biases exist regarding sample submissions. It is possible that our findings may have overestimated the true prevalence of MDR *Salmonella* among clinical isolates of swine populations in Thailand. Nevertheless, our approach provides an early warning for emerging pathogens and antimicrobial resistance threats in Thailand, and the results provide valuable information for managing *Salmonella* in swine.

## Conclusion

Our results reveal the prevalence and resistance characteristics of *Salmonella* serovars in clinical swine samples for the first time in Thailand. Choleraesuis was the most predominant serovar followed by monophasic Typhimurium, Typhimurium, and Rissen. All isolates were MDR and collectively yielded 29 distinct MDR patterns. These results raise the alarm for the need to develop appropriate therapeutic treatments and call attention on the reasonable use of antimicrobials. The high rate of resistance to critical antimicrobials such as CAZ, CRO, and CTX and the reduced susceptibility to CIP among screened *Salmonella* serovars provide additional evidence on the possibility that such resistant strains may disseminate through the food supply chain, leading to therapeutic failures in food animals and humans. Finally, this study provides profiles of multidrug resistance among *Salmonella* serovars in clinically ill swine. These results provide useful information to improve strategies to control and treat *Salmonella* infections in swine populations in Thailand.

## Authors’ Contributions

SK designed the study, analyzed and interpreted the data, and drafted and revised the manuscript. SC performed the experiments and contributed reagents/materials/analysis tools. DS and PN performed the experiments and collected the data. All authors have read and approved the final manuscript.
